# Continuous Writing, Reviewing, and Editing by Physicians

**DOI:** 10.31662/jmaj.2023-0115

**Published:** 2023-11-16

**Authors:** Soichiro Saeki

**Affiliations:** 1 Center Hospital of the National Center for Global Health and Medicine, Tokyo, Japan

**Keywords:** continuing medical education, case report, letter to the editor, undergraduate, postgraduate

The author extends his gratitude to Matsubara and Lefor for their interest in author’s preceding manuscript ^(1), (2)^. Their insight on involving the faculty members in writing the case reports warrants special attention, as only a limited number of manuscripts emphasize the significance of writing for senior staff members. Their manuscript elucidated a crucial point; as clinicians in Japan, we believed that case reports should be exclusively written by young trainees or students rather than by esteemed senior staff.

As the Japanese are generally perceived as more introverted than people from the western nations ^(3)^, promoting the continuous dissemination of research through staff physicians’ paper writing can prove advantageous in overcoming such a context. Particularly, it is advisable for faculties such as non-university hospital healthcare institutions, whose primary focus is not centered on academic accomplishments, to consistently engage in the writing process.

To facilitate the composition of papers for noteworthy cases or research findings that merit transformation into published works, ongoing training is imperative. One approach, is to continuously write concise manuscripts, such as letters to the editor ^(2)^. This practice fosters a deeper contemplation of literature and remains valuable as a method for continuing medical education. Another worthwhile method is active participation in peer review and editorial tasks. Peer review, similar to letter writing, necessitates critical evaluation of manuscripts. Notably, there is a paucity of reviewers from countries beyond Europe and North America ^(4)^, and engaging in peer review from Japan can contribute to enhancing Japan’s prominence within scientific societies. Even individuals in the early stages of their careers can acquire a broad range of scientific knowledge through peer review and editorial tasks ^(5)^, thereby gaining exposure to cutting-edge research, fostering professional advancement, and ultimately making contributions to the scientific community ^(4), (5)^.

However, such opportunities are not guaranteed. While authoring manuscripts is typically a proactive endeavor, reviewing and editing necessitates passive invitations from the editorial boards of academic journals. Although it may be difficult for journal editors to enlist researchers with limited research experience, efforts to broaden the pool of editors and reviewers by actively recruiting a diverse range of individuals should be viewed favorably.

The pivotal factor in academic paper writing is continuity, regardless of one’s seniority ^(1)^. It is commendable for each department to actively engage in various aspects of scientific publications as it nurtures a spirit of amicable competition and improvement.

## Article Information

### Conflicts of Interest

None

### Acknowledgement

The author thanks all the colleagues for helpful discussions on this topic. The author acknowledges the use of Microsoft Bing (Microsoft Corporation, Redmond, WA, USA) and ChatGPT (OpenAI, L.L.C., San Francisco, CA, USA) for primary language editing. The views expressed in this manuscript are those of the author and do not necessarily represent the author’s institutions.

### Author Contributions

The author is solely responsible for the manuscript content. Artificial intelligence technology was used for language editing process, and such content was reviewed by the author. The author’s institution played no role in the conceptualization of this manuscript.

### Approval by Institutional Review Board (IRB)

Not applicable.
